# Stacking of a low-lignin trait with an increased guaiacyl and 5-hydroxyguaiacyl unit trait leads to additive and synergistic effects on saccharification efficiency in *Arabidopsis thaliana*

**DOI:** 10.1186/s13068-018-1257-y

**Published:** 2018-09-20

**Authors:** Lisanne de Vries, Ruben Vanholme, Rebecca Van Acker, Barbara De Meester, Lisa Sundin, Wout Boerjan

**Affiliations:** 1 Department of Plant Biotechnology and Bioinformatics, Ghent University, Technologiepark 927, 9052 Ghent, Belgium; 2 VIB Center for Plant Systems Biology, Technologiepark 927, 9052 Ghent, Belgium

**Keywords:** Saccharification, Lignin, Gene stacking, *Transaldolase* (*tra*), *Cinnamate 4*-*hydroxylase* (*c4h*), *4*-*coumarate:CoA ligase* (*4cl*), *Caffeic acid O*-*methyltransferase* (*comt*), *Arabidopsis thaliana*

## Abstract

**Background:**

Lignocellulosic biomass, such as wood and straw, is an interesting feedstock for the production of fermentable sugars. However, mainly due to the presence of lignin, this type of biomass is recalcitrant to saccharification. In Arabidopsis, lignocellulosic biomass with a lower lignin content or with lignin with an increased fraction of guaiacyl (G) and 5-hydroxyguaiacyl (5H) units shows an increased saccharification efficiency. Here, we stacked these two traits and studied the effect on the saccharification efficiency and biomass yield, by combining either *transaldolase* (*tra2*), *cinnamate 4*-*hydroxylase* (*c4h*-*3*), or *4*-*coumarate:CoA ligase* (*4cl1*-*1*) with *caffeic acid O*-*methyltransferase* (*comt*-*1* or *comt*-*4*) mutants.

**Results:**

The three double mutants (*tra2 comt*-*1*, *c4h*-*3 comt*-*4,* and *4cl1*-*1 comt*-*4*) had a decreased lignin amount and an increase in G and 5H units in the lignin polymer compared to wild-type (WT) plants. The *tra2 comt*-*1* double mutant had a better saccharification efficiency compared to the parental lines when an acid or alkaline pretreatment was used. For the double mutants, *c4h*-*3 comt*-*4* and *4cl1*-*1 comt*-*4*, the saccharification efficiency was significantly higher compared to WT and its parental lines, independent of the pretreatment used. When no pretreatment was used, the saccharification efficiency increased even synergistically for these mutants.

**Conclusion:**

Our results show that saccharification efficiency can be improved by combining two different mutant lignin traits, leading to plants with an even higher saccharification efficiency, without having a yield reduction of the primary inflorescence stem. This approach can help improve saccharification efficiency in bio-energy crops.

**Electronic supplementary material:**

The online version of this article (10.1186/s13068-018-1257-y) contains supplementary material, which is available to authorized users.

## Background

Lignocellulosic biomass is an abundant renewable feedstock, with a high potential for the production of bio-based chemicals. As such, this type of biomass has the potential to play an essential role in the shift from the current fossil-based economy towards a bio-based economy [1, 2]. Lignocellulosic biomass consists predominantly of secondary thickened plant cell walls, which are mainly composed of the polysaccharides cellulose and hemicellulose and the phenolic polymer lignin [3]. Lignin provides the plant with strength and rigidity, facilitates upward water transport in the vessels, and forms a barrier to pathogens [4–6]. The presence of lignin is also a major reason why lignocellulosic biomass is recalcitrant to enzymatic saccharification (i.e., the hydrolysis of the plant polysaccharides into fermentable monosaccharides) [7–10], because it covers the cellulose microfibrils and thereby prevents the cellulases from accessing the cellulose surface [11]. Moreover, the saccharification enzymes are unspecifically adsorbed onto the lignin polymer [7]. To overcome the recalcitrance of the plant cell wall and increase the saccharification efficiency, strategies aiming at lowering the amount or changing the composition of lignin have been tested in various plant species with some degree of success [8–10, 12–17]. However, these alterations are often accompanied by a biomass penalty, hindering their potential for applications. Therefore, there is a need to optimize the balance between lignin engineering, yield penalty, and saccharification efficiency.

**Fig. 1:**
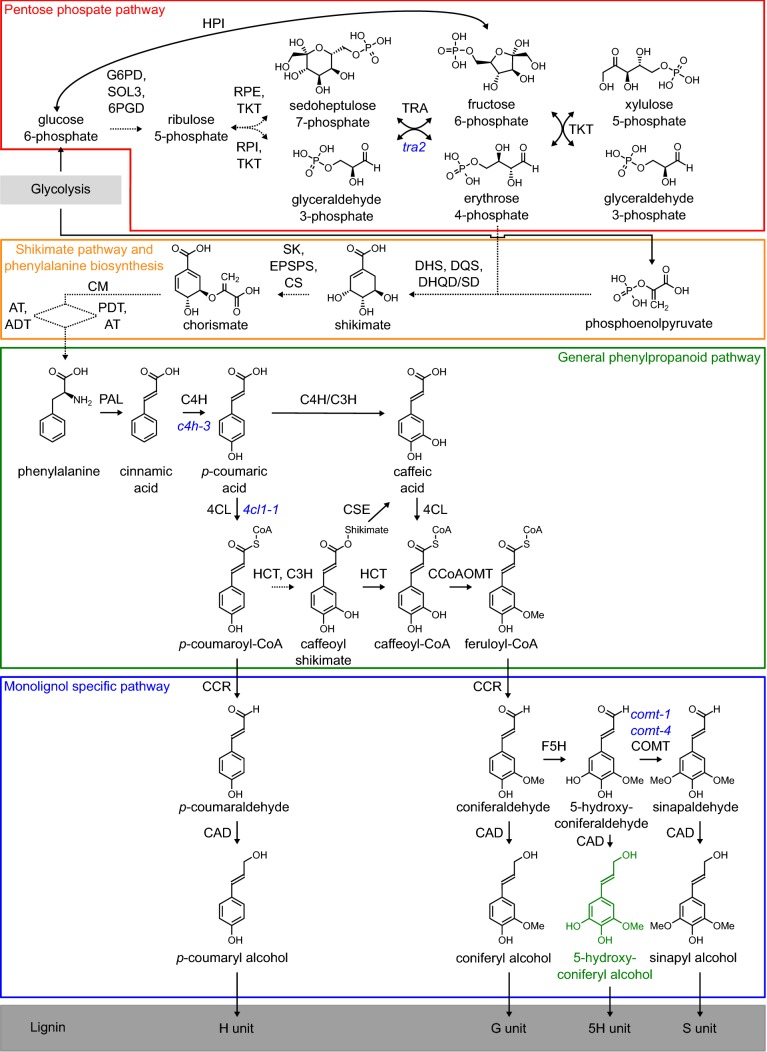
Lignin biosynthesis. The red box shows the pentose phosphate pathway, the orange box shows the shikimate pathway and the synthesis of the aromatic amino acid phenylalanine, the green box shows the general phenylpropanoid pathway and the blue box shows the monolignol specific pathway. Dotted arrows indicate more than one (enzymatic) conversion. In blue are the mutant alleles studied, in green 5-hydroxyconiferyl alcohol, that upon incorporation in lignin, gives rise to 5-hydroxyguaiacyl units. *4CL* 4-coumarate:CoA ligase, *6PGD* 6-phosphogluconate dehydrogenase, *ADT* arogenate dehydratase, *AT* amino transferase, *C3H* P-coumarate 3-hydroxylase, *C4H* cinnamate 4-hydroxylase, *CAD* cinnamyl alcohol dehydrogenase, *CCoAOMT* caffeoyl-COA *O*-methyltransferase, *CCR* cinnamoyl-CoA reductase, *CM* chorismate mutase, *COMT* caffeic acid *O*-methyltransferase, *CS* chorismate synthase, *CSE* caffeoyl shikimate esterase, *DHQD/SD* 3-dehydroquinate dehydratase/shikimate dehydrogenase, *DHS* 3-deoxy-d-arabino-heptulosonate 7-phosphate synthase, *DQS* 3-dehydroquinate synthase, *EPSPS* 5-enolpyruvylshikimate-3-phosphate synthase, *F5H* ferulate 5-hydroxylase, *G6PD* glucose-6-phosphate dehydrogenase, *HCT* hydroxycinnamoyl-COA shikimate/quinate hydroxycinnamoyl transferase, *HPI* hexose phosphate isomerase, *PAL* penylalanine ammonia-lysase, *PDT* prephenate dehydratase, *RPE* ribose-5-phosphate epimerase, *RPI* ribose-5-phosphate isomerase, *SK* shikimate kinase, *SOL3* 6-gluconolactonase, *TKT* transketolase, *TRA* transaldolase

In dicotyledonous plants, lignin is predominantly composed of the traditional monolignols coniferyl alcohol and sinapyl alcohol and traces of *p*-coumaryl alcohol. These monolignols are synthesized from the aromatic amino acid phenylalanine via the general phenylpropanoid and monolignol-specific pathways (Fig. 1) [18, 19]. Phenylalanine is synthesized via the plastid-localized shikimate pathway, which converts erythrose 4-phosphate and phosphoenolpyruvate into chorismate [20]. Erythrose 4-phosphate and phosphoenolpyruvate are derived from the pentose phosphate pathway and glycolysis, respectively [21, 22]. The first step in the general phenylpropanoid pathway is the deamination of phenylalanine to produce cinnamic acid, which is followed by several hydroxylation and *O*-methylation steps of the aromatic ring and a multi-step conversion of the γ-carboxylic acid to an alcohol, eventually resulting in the traditional monolignols (Fig. 1). After their biosynthesis, the monolignols are transported to the cell wall, where they are oxidized to monolignol radicals by laccases and peroxidases [23, 24]. These radicals couple in a combinatorial fashion, generating the lignin polymer. Coniferyl alcohol, sinapyl alcohol, and *p*-coumaryl alcohol give rise to guaiacyl (G), syringyl (S), and *p*-hydroxyphenyl (H) units, respectively. Most of these units are linked via β-*O*-4 (ether linkages), β–β, or β-5 structures (both carbon–carbon linkages) [18, 19, 25]. Due to the nature of this chemical coupling process, a whole series of additional phenolic compounds has the capacity to be incorporated into the lignin polymer [26]. These compounds can be pathway intermediates or derivatives thereof, such as ferulic acid and 5-hydroxyconiferyl alcohol, as well as monomers generated via genetic engineering, such as ferulate conjugates [14, 27, 28].

Lignin amount and composition can be engineered by perturbing the flux through the biosynthetic pathways involved in monolignol biosynthesis [27, 28]. For instance, perturbing caffeic acid *O*-methyltransferase (COMT) activity in Arabidopsis, poplar, maize, alfalfa, sorghum, *Brachypodium,* and sugarcane results in lignin with an increase in G units, a decrease in S units, and the appearance of 5-hydroxyguaiacyl (5H) units derived from the incorporation of 5-hydroxyconiferyl alcohol and 5-hydroxyconiferaldehyde [8, 29–38]. The polymerization of 5H units leads to benzodioxane structures in the lignin polymer, thereby altering its structural and physico-chemical properties [8, 35, 39]. Because of this altered lignin structure and/or the increased incorporation of G units in the lignin polymer, the saccharification efficiency is improved with 56% in Arabidopsis *comt* mutants [8], as is the case in *Brachypodium* and sugarcane plants defective in *COMT* [36, 37]. Nevertheless, this increase might not only be a result of the altered lignin structure, but also of the slightly reduced lignin content that is observed additionally in these mutants [36, 37].

Plants with less lignin can be engineered by reducing the expression of, for instance, *transaldolase* (*TRA*), *cinnamate 4*-*hydroxylase* (*C4H*), or *4*-*coumarate:CoA ligase* (*4CL*). TRA2 is an enzyme in the pentose phosphate pathway, responsible for the conversion of sedoheptulose 7-phosphate and glyceraldehyde 3-phosphate to erythrose 4-phosphate and fructose 6-phosphate (Fig. 1) [40]. The Arabidopsis *tra2* mutant has a reduction of 15% in lignin amount and an increased S/G ratio with no measurable effect on biomass yield [41]. C4H is involved in the second step of the phenylpropanoid pathway, i.e., the conversion of *trans*-cinnamic acid to *p*-coumaric acid (Fig. 1). A full knockout of *C4H* in Arabidopsis results in a seedling lethal phenotype [42]. However, the leaky allele *c4h*-*3* [also named *reduced epidermal fluorescence 3* (*ref3*-*3*)] has a point mutation that results in 35% less lignin deposition, an increased S/G ratio, and a higher saccharification efficiency, without an effect on biomass yield [8, 41, 42]. In biomass crops poplar, alfalfa, and eucalyptus, downregulation of *C4H* also resulted in a reduced amount of lignin [43–45]. In *C4H* downregulated poplar, no significant change in S/G ratio was observed and there was a slight reduction in height as compared to WT [44]. In contrast, in alfalfa and eucalyptus, both a reduction in height and a reduced S/G ratio were observed for the *C4H*-downregulated plants [43, 45]. 4CL1 catalyzes the conversion of *p*-coumaric acid to *p*-coumaryl-CoA (Fig. 1) [46, 47]. The Arabidopsis *4cl1*-*1* mutant has a 26% reduction in lignin amount, an increased S/G ratio, and a higher saccharification efficiency as compared to WT plants and did not have a biomass yield penalty [8]. A reduced expression of *4CL* in sugarcane, and the mutation of *4CL* in poplar and switchgrass (both induced by CRISPR/Cas9) also resulted in less lignin in the cell wall [48–50]. For sugarcane and switchgrass, an increased S/G ratio and an increased saccharification efficiency compared to the WT were observed, while for poplar, the S/G ratio did not differ and the saccharification efficiency was not improved [49–52].

Considering that both strategies, i.e., engineering lignin monomeric composition and lignin amount, have been shown to improve saccharification efficiency individually, it is reasonable to hypothesize that stacking these traits could result in even larger improvements. However, combining different lignin mutations may lead to a biomass yield penalty, which makes these plants less promising as biomass resource for the bio-refinery. Indeed, several attempts to stack multiple lignin traits have already been described. In Arabidopsis, stacking of *cinnamyl alcohol dehydrogenase*-*c* (*cad*-*c*) and *cad*-*d* mutations that enrich the lignin in coniferaldehyde and sinapaldehyde with *ferulate 5*-*hydroxylase* (*F5H*) overexpression that enriched the lignin in S units, resulted in dwarfed plants, whereas the *cad*-*c cad*-*d* and *F5H* overexpressing parental lines did not have an obvious yield penalty [53]. The authors hypothesized that the biomass yield penalty was due to deficiencies in the production of dehydrodiconiferyl alcohol glucosides, CAD-dependent compounds that might play a role in cell expansion and growth, or due to the change in transcription caused by changes in the flux through the phenylpropanoid pathway [53]. On the other hand, crossing *cad*-*c cad*-*d* with *f5h* [also named *ferulic acid 5*-*hydroxylase1* (*fah1*)], which is enriched in G units, did not result in a biomass yield penalty. Nevertheless, the saccharification efficiency of the *fah1 cad*-*c cad*-*d* triple mutant did not improve compared to that of the *cad*-*c cad*-*d* parental line. In both gene-stacking combinations, the lignin polymers were enriched in cinnamaldehydes [53]. Stacking *cad*-*c cad*-*d* with *cinnamoyl*-*CoA reductase 1* (*ccr1*) in Arabidopsis resulted in plants with a severe dwarf phenotype (more severe than the parental *ccr1* line), thought to be due to higher amounts of flavonol glycosides, sinapoyl malate, and feruloyl malate compared to the individual mutants, and male sterility due to the lack of lignification in the anther endothecium [54]. Stacking of *comt* with *F5H* overexpression resulted in a strong enrichment in benzodioxane units in lignin, male sterility, and growth defects in Arabidopsis [55, 56]. Stacking of *caffeoyl*-*CoA O*-*methyltransferase1* (*ccoaomt1*) with *comt* in Arabidopsis did not result in viable plants; the development of *ccoaomt1 comt* was arrested at the seedling stage [57], presumably because CCoAOMT1 and COMT act redundantly in the conversion of caffeoyl-CoA to feruloyl-CoA (Fig. 1). Hence, stacking different lignin mutations does not by definition result in healthy plants with an increased saccharification efficiency. Nevertheless, the use of gene stacking can improve the quality of lignocellulosic biomass as demonstrated by stacking the overexpression of *UDP*-*glucose 4*-*epimerase2* (*UGE2*) and *galactan synthase 1* (*GalS1*), two genes involved in the biosynthesis of hemicellulose [58]. The *UGE2 GalS1* overexpressing plants had a higher hexose/pentose ratio than plants overexpressing *UGE2* or *GalS1* alone. Because hexoses are more easily fermented by yeast than pentoses are, an increased content of hexose in biomass crops is of interest for bio-refinery purposes.

Here, we studied the effects of stacking a mutation in *TRA2*, *C4H,* or *4CL1* with a mutation in *COMT* on the saccharification and biomass yield in Arabidopsis. We combined the low-lignin trait of either *tra2*, *c4h*-*3,* or *4cl1*-*1* mutants with the G- and 5H-rich lignin trait of *comt*-*1* or *comt*-*4* mutants. We explicitly chose these lignin mutants as parental lines as they have an increase in saccharification efficiency, without having a yield penalty. For *tra2*, *c4h*-*3,* and *4cl1*-*1,* this increase in saccharification efficiency is caused by the reduction in lignin amount, while for *comt,* this increase is caused by a change in lignin composition, according to saccharification models [8]. We show that additive and synergistic effects on saccharification efficiency can be achieved by gene stacking without causing a yield penalty. The increased saccharification efficiency was most pronounced when no pretreatment was used, which makes this gene-stacking strategy a promising approach for translation to bio-refinery crops.

## Results

### Stacking reduced lignin content with G- and 5H-rich lignin does not result in a biomass yield penalty

In an attempt to stack a low-lignin trait with an increased incorporation of G and 5H units in Arabidopsis, we crossed either *tra2*, *c4h*-*3,* or *4cl1*-*1* with either *comt*-*1* or *comt*-*4*. To study the effects of these gene stackings on cell-wall characteristics and biomass yield, the three resulting homozygous double mutants, *tra2 comt*-*1*, *c4h*-*3 comt*-*4,* and *4cl1*-*1 comt*-*4*, were grown with their respective control lines (wild type and their respective parental lines) in three independent experiments. Because lignin perturbations often have negative repercussions on biomass yield, we first determined the height and dry weight of the senesced primary inflorescence stems of the double mutants as a measure for their biomass (Table 1). The height of the WT primary inflorescence stems varied between 46.8 and 51.3 cm and the mass ranged between 43.1 and 53.5 mg. Similar values for height and mass were obtained for the double mutants *tra2 comt*-*1*, *c4h*-*3 comt*-*4,* and *4cl1*-*1 comt*-*4* and their respective parents (Table 1). It is important to notice that there were slight differences between genotypes grown in different experiments, but there are no differences between the genotypes within a given experiment. Next, the cell-wall residue (CWR) of the primary inflorescence stem biomass was determined by removing the soluble molecules via sequential extraction. The CWR of the WT varied between 74.9 and 77.8% of the dry weight. Similar values were obtained for the three double mutants *tra2 comt*-*1*, *c4h*-*3 comt*-*4,* and *4cl1*-*1 comt*-*4* and their respective parental lines (Table 1).

**Table 1:** Phenotypic traits

Line	Height (cm)	Mass (mg)	% CWR
*tra2 comt*-*1*			
WT	46.8 ± 5.3	43.1 ± 9.9	75.4 ± 1.7
*tra2*	46.5 ± 4.7	37.5 ± 11.3	75.2 ± 1.1
*comt*-*1*	48.3 ± 4.6	44.4 ± 11.3	74.5 ± 2.1
*tra2 comt*-*1*	48.7 ± 3.8	42.6 ± 10.8	74.0 ± 2.2
*c4h*-*3 comt*-*4*			
WT	48.2 ± 4.9	53.5 ± 15.0	74.9 ± 1.8
*c4h*-*3*	50.5 ± 3.3	55.8 ± 12.9	76.0 ± 2.5
*comt*-*4*	50.5 ± 5.6	54.9 ± 9.5	75.7 ± 2.3
*c4h*-*3 comt*-*4*	50.2 ± 4.1	54.8 ± 12.3	74.2 ± 1.4
*4cl1*-*1 comt*-*4*			
WT	51.3 ± 4.0	43.7 ± 7.7	77.8 ± 1.2
*4cl1*-*1*	50.2 ± 3.8	43.6 ± 7.5	77.0 ± 1.7
*comt*-*4*	52.5 ± 3.6	43.9 ± 7.1	76.9 ± 1.4
*4cl1*-*1 comt*-*4*	51.6 ± 3.0	43.9 ± 6.6	77.1 ± 1.4

Height and mass were determined on fully senesced primary inflorescence stems of the three double mutants, wild type (WT), and the corresponding parental lines. The data represent the average ± standard deviation of at least 24 replicates per line. The cell-wall residue (CWR) was determined gravimetrically after extraction and is expressed in % dry weight ± standard deviation (*n* = 10). No significant differences were observed at the 0.01 significance level (ANOVA, pairwise comparison with Holm correction)

*WT* wild type

To conclude, no biomass yield penalty was observed for the double mutants and their parental lines, and all lines and the WT had a similar CWR.

### Combining low lignin with the G- and 5H-rich trait results in a reduced lignin content and changed lignin composition in the double mutants

We determined the lignin amount of the senesced primary inflorescence stems via the spectrophotometrical acetyl bromide (AcBr) method. Compared to WT stems, the AcBr lignin content of *tra2 comt*-*1*, *c4h*-*3 comt*-*4,* and *4cl1*-*1 comt*-*4* stems showed a significant decrease of 15%, 41%, and 31%, respectively (Table 2). The AcBr lignin content of stems of *tra2 comt*-*1* double mutants was not significantly different from that of its parental lines (*tra2* and *comt*-*1*). Stems of the *c4h*-*3 comt*-*4* double mutant showed no significant differences in lignin content compared to that of its *c4h*-*3* parental mutant line, but had a significant lignin reduction of 32% compared to the parental *comt*-*4* mutant. The stems of *4cl1*-*1 comt*-*4* had a significant decrease in lignin of 16% and 32% compared to those of the *4cl1*-*1* and *comt*-*4* parental lines, respectively (Table 2). Notably, in the experiment with the *4cl1*-*1 comt*-*4* double mutant, the *comt*-*4* parental line had a reduced lignin amount as compared to wild type. A reduction in lignin amount was not observed in Arabidopsis *comt* mutants in the experiments with *tra2 comt*-*1* and *c4h*-*3 comt*-*4* double mutants (Table 1) nor in previous reports [8]. Because the *comt*-*4* genotype was used in many of the experiments, the differences in lignin amount in the independent experiments must be caused by—yet unknown—subtle differences in growth conditions. Furthermore, the *4cl1*-*1 comt*-*4* double mutant had less lignin than the *4cl1*-*1* low-lignin parental line; the reduction in lignin in *4cl1*-*1* and *comt*-*4* mutants appeared additive.

**Table 2:** Lignin amount and composition

Line	AcBr	S/G	% H	% G	% S	% 5H
*tra2 comt*-*1*						
WT	11.2 ± 0.9^a^	0.63 ± 0.08^a^	0.4 ± 0.1^a^	60.9 ± 2.9^a^	38.5 ± 2.9^a^	0.24 ± 0.05^a^
*tra2*	10.0 ± 1.4^ab^*	0.79 ± 0.11^b^	0.6 ± 0.2^a^	55.4 ± 3.3^b^	43.7 ± 3.3^a^	0.27 ± 0.05^a^
*comt*-*1*	10.9 ± 0.7^ab^	0.02 ± 0.01^c^	0.5 ± 0.2^a^	96.9 ± 1.4^c^	1.5 ± 1.4^b^	0.99 ± 0.37^b^
*tra2 comt*-*1*	9.6 ± 1.0^b^	0.01 ± 0.00^c^	0.7 ± 0.2^a^	96.8 ± 0.4^c^	1.4 ± 0.5^b^	0.99 ± 0.29^b^
*c4h*-*3 comt*-*4*						
WT	11.3 ± 0.8^a^	0.52 ± 0.03^a^	0.7 ± 0.1^a^	65.2 ± 1.2^a^	33.9 ± 1.2^a^	0.16 ± 0.02^a^
*c4h*-*3*	8.1 ± 0.8^b^	1.05 ± 0.07^b^	1.2 ± 0.4^b.c^	48.2 ± 1.6^b^	50.5 ± 1.9^b^	0.18 ± 0.01^b^
*comt*-*4*	11.6 ± 1.9^a^	0.06 ± 0.01^c^	1.0 ± 0.3^a.b^	92.2 ± 1.0^c^	5.6 ± 0.8^c^	1.25 ± 0.14^c^
*c4h*-*3 comt*-*4*	7.9 ± 0.3^b^	0.13 ± 0.01^d^	1.5 ± 0.2^c^	85.4 ± 0.8^d^	11.5 ± 0.8^d^	1.54 ± 0.39^c^
*4cl1*-*1 comt*-*4*						
WT	11.8 ± 0.7^a^	0.62 ± 0.18^a^	1.2 ± 0.2^a^	61.7 ± 6.9^a^	36.9 ± 6.8^a^	0.16 ± 0.04^a^
*4cl1*-*1*	8.3 ± 1.4^b^	0.90 ± 0.04^b^	2.0 ± 0.3^b, c^	51.7 ± 1.2^b^	46.2 ± 1.2^b^	0.16 ± 0.02^a^
*comt*-*4*	10.2 ± 1.0^c^	0.11 ± 0.02^c^	1.6 ± 0.5^a, c^	87.2 ± 2.3^c^	9.8 ± 1.9^c^	1.49 ± 0.55^b^
*4cl1*-*1 comt*-*4*	6.9 ± 0.5^d^	0.18 ± 0.02^d^	2.4 ± 0.6^b^	82.0 ± 1.6^d^	14.5 ± 1.4^d^	1.08 ± 0.39^b^

Lignin amount and composition of the three double mutants, wild type (WT), and the corresponding parental lines (*n* = 10) (± SD). The lignin amount was determined via the AcBr method and is expressed as % CWR. The lignin composition was determined via thioacidolysis. The relative amounts of the different lignin units were calculated based on the total thioacidolysis yield. The S/G ratio was calculated based on the absolute values for S and G (expressed in μmol mg^−1^ AcBr lignin). Significance groups are indicated with the same letter in superscript and different letters represent significant differences at the 0.01 significance level (ANOVA or Kruskal–Wallis, pairwise comparison with Holm correction). *p value 0.051 after pairwise comparison with Holm correction

*WT* wild type

The lignin composition was analyzed via thioacidolysis, which results in the release of lignin monomers involved in β-*O*-4-ether bonds that can be analyzed with gas chromatography–mass spectrometry (GC–MS). Lignin in angiosperms is mainly composed of G and S units. Therefore, the S/G ratio is commonly used to describe the lignin composition. The G and S units accounted for about 99% of the total released monomers in the WT plants and in the single and double mutants. The S/G ratios were significantly increased in the parental lines *tra2*, *c4h*-*3,* and *4cl1*-*1* and reduced in *comt*-*1* and *comt*-*4*, consistent with the previous report [8]. In the *tra2 comt*-*1*, *c4h*-*3 comt*-*4,* and *4cl1*-*1 comt*-*4* double mutants, the S/G ratio was significantly lower than that in the WT and also significantly lower than that in the low-lignin *tra2*, *c4h*-*3,* and *4cl1*-*1* parental lines (Table 2). There were no significant differences in S/G ratio between the *tra2 comt*-*1* double mutant and its parental line *comt*-*1*. However, the S/G ratio for *4cl1*-*1 comt*-*4,* and *c4h*-*3 comt*-*4* was significantly higher compared to that of their parental *comt*-*4* line. The relative frequency of H units in *c4h*-*3 comt*-*4* and *4cl1*-*1 comt*-*4* double mutants was significantly higher than that in the WT but similar to that of their corresponding low-lignin parental lines *c4h*-*3* and *4cl1*-*1* (Table 2). Consistent with the previous research [8], the relative amount of 5H units was increased in the *comt*-*1* and *comt*-*4* parental lines compared to the WT. This increase in relative amount of 5H units was also found in the lignin polymer of *tra2 comt*-*1*, *c4h*-*3 comt*-*4,* and *4cl1*-*1 comt*-*4* compared to the WT and their parental *tra2*, *c4h*-*3,* and *4cl1*-*1* lines, respectively.

In conclusion, although, as anticipated, all double mutants had a significant reduction in lignin amount compared to the WT, there were differences between the three double mutants when comparing them with their corresponding parents: *tra2 comt*-*1* had the same amount of lignin compared to both its parental lines, whereas *c4h*-*3 comt*-*4* had a lower lignin amount compared to *comt*-*4* and *4cl1*-*1 comt*-*4* had a lower amount of lignin compared to either of its parental lines. The double mutants displayed a lower S/G ratio and an increase in 5H units compared to the WT.

### Stacking reduced lignin content with G- and 5H-rich lignin has synergistic effects on the saccharification efficiency

To calculate the saccharification efficiency, we first determined the cellulose amount. After an extraction with trifluoroacetic acid (TFA) to remove the hemicellulose and amorphous cellulose, the amount of crystalline cellulose in the cell wall was spectrophotometrically determined via the Updegraff method (Table 3). The *tra2 comt*-*1* double mutant and the *4cl1*-*1 comt*-*4* double mutant had 38.7% and 39.3% of cellulose/CWR, respectively, which did not differ significantly from the WT and their parental lines. However, senesced stems of the *c4h*-*3 comt*-*4* double mutant consisted of 40% cellulose/CWR, a 7% increase compared to the WT. Compared to its parental lines, *c4h*-*3* and *comt*-*4*, the double mutant *c4h*-*3 comt*-*4* had 3% and 5% more cellulose/CWR, respectively.

**Table 3:** Cellulose amount

Plant	Cellulose (% CWR)
*tra2 comt*-*1*	
WT	37.3 ± 0.9^a^
*tra2*	38.2 ± 0.6^a^
*comt*-*1*	38.1 ± 1.3^a^
*tra2 comt*-*1*	38.7 ± 0.9^a^
*c4h*-*3 comt*-*4*	
WT	37.1 ± 0.6^a^
*c4h*-*3*	38.7 ± 1.1^b^
*comt*-*4*	38.2 ± 0.9^a, b^
*c4h*-*3 comt*-*4*	40.0 ± 0.6^c^
*4cl1*-*1 comt*-*4*	
WT	38.2 ± 0.6^a^
*4cl1*-*1*	39.2 ± 1.0^a^
*comt*-*4*	39.3 ± 1.0^a^
*4cl1*-*1 comt*-*4*	39.3 ± 0.7^a^

The crystalline cellulose amount was determined with the Updegraff cellulose assay. Measured amounts were normalized to the CWR. Values denote average ± SD (*n* = 10). Significance groups are indicated with the same letter in superscript and different letters represent significant differences at the 0.01 significance level (ANOVA, pairwise comparison with Holm correction)

*WT* wild type

To investigate the effect of stacking *tra2*, *c4h*-*3,* and *4cl1*-*1* mutants with the *comt* mutants on the saccharification efficiency, a saccharification protocol for small-scale lignocellulosic biomass samples was applied as previously described [8, 59]. Three experimental setups were used, without pretreatment, and with acid or alkaline pretreatment. In each setup, the saccharification of the senesced primary inflorescence stems was measured at 6, 24, 48, and 72 and, contingent on not reaching a plateau, 96 h. The plateau was defined as the timepoint at which the absolute increase in cellulose-to-glucose conversion was smaller than 2% in 24 h. Depending on the setup and pretreatment used, this plateau was reached at 72 or 96 h (Additional files 1 and 2).

**Fig. 2:**
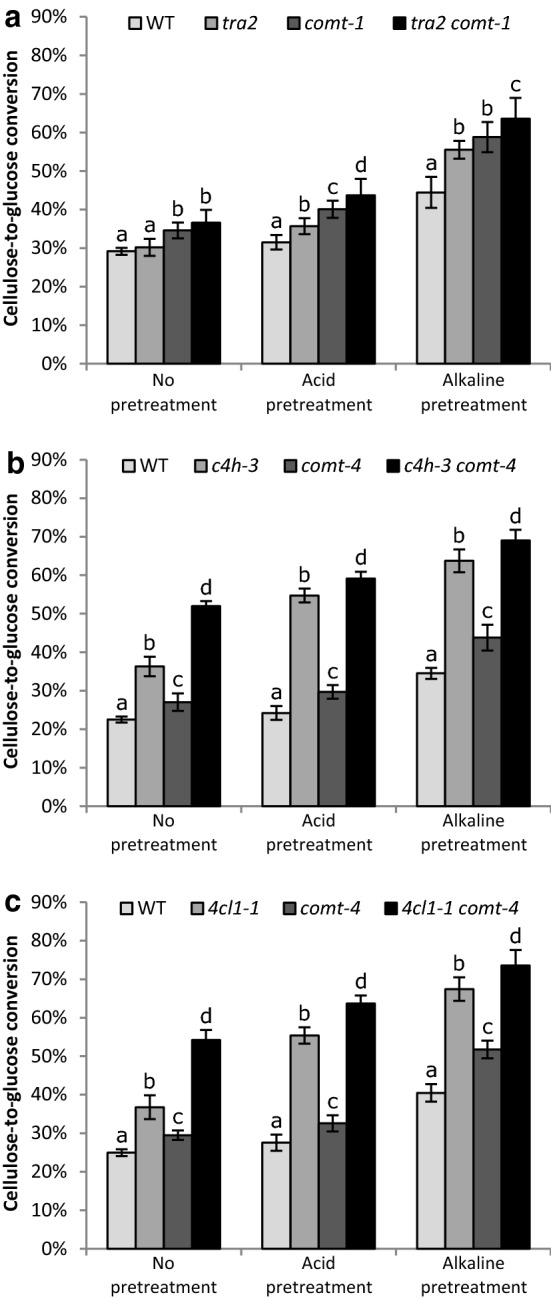
Cellulose-to-glucose conversions. Cellulose-to-glucose conversions of the *tra2 comt*-*1*, *c4h*-*3 comt*-*4,* and *4cl1*-*1 comt*-*4* double mutants, wild type, and the corresponding parental lines, without, with acid, and with alkaline pretreatment. The conversions were calculated based on the saccharification efficiency and cellulose content (both on CWR basis) and are expressed in % converted cellulose at the moment the plateau was reached. **a** Cellulose-to-glucose conversion for *tra2 comt*-*1* and its respective control lines. **b** Cellulose-to-glucose conversion for *c4h*-*3 comt*-*4* and its respective control lines. **c** Cellulose-to-glucose conversion for *4cl1*-*1 comt*-*4* and its respective control lines. The error bars represent standard deviations (*n* = 10). Significance is presented as significance groups, and values with the same letter do not differ significantly from each other at the 0.05 significance level (ANOVA, pairwise comparison Holm correction)

At the plateau, the *tra2 comt*-*1* double mutant had a relative increase in saccharification efficiency of 25%, 39%, and 41% without, with an acid and with an alkaline pretreatment, respectively, compared to the WT (Fig. 2a, Additional file 3). With acid or alkaline pretreatment, *tra2 comt*-*1* showed a significantly higher saccharification efficiency compared to that of either parental lines, and the effect was additive (Additional file 3). With an acid pretreatment, the saccharification efficiency of *tra2 comt*-*1* was increased by 22% and 9% compared to *tra2* and *comt*-*1*, respectively. With an alkaline pretreatment, the saccharification efficiency of *tra2 comt*-*1* was increased by 15% and 8% compared to *tra2* and *comt*-*1,* respectively. If the glucose release is calculated per CWR, similar results were obtained (Additional files 4, 5, 6). The primary inflorescence stem pieces started to degrade into smaller fibers for *tra2* and *tra2 comt*-*1* under alkaline condition (Additional file 7).

Independent of the pretreatment, *c4h*-*3 comt*-*4* had a better saccharification efficiency than the WT and either of the parental *c4h*-*3* and *comt*-*4* lines (Fig. 2b). Compared to the WT, the saccharification efficiency was increased by 131%, 144%, and 100% when no, an acid or an alkaline pretreatment was used, respectively. A synergistic effect was observed when no pretreatment was used, and an additive effect when an acid or alkaline pretreatment was used (Additional file 3). Compared to the parental lines *c4h*-*3* and *comt*-*4*, an increase of 43% and 92%, respectively, was observed in the double mutant when no pretreatment was used. When an acid pretreatment was used, an increase of 8% and 99% was observed in the double mutant compared to *c4h*-*3* and *comt*-*4*, respectively. When an alkaline pretreatment was used, the increase was 8% and 58%, respectively. If the glucose release is calculated per CWR, similar results were obtained (Additional files 4, 5, 6). In addition, here, degradation of the primary inflorescence stem pieces into fibers was clearly observed under acid and alkaline pretreatment for *c4h*-*3* and *c4h*-*3 comt*-*4* (Additional file 7).

In addition, for *4cl1*-*1 comt*-*4*, a better saccharification efficiency was observed compared to the WT and either of the parental lines, independent of the pretreatment used (Fig. 2c). The saccharification efficiency of *4cl1*-*1 comt*-*4* was increased by 117%, 131%, and 82% compared to that of the WT when no, an acid or an alkaline pretreatment was used, respectively. The stacking of the *4cl1*-*1* and *comt*-*4* mutations led to a synergistic effect on the saccharification efficiency when the biomass was not pretreated, with a relative increase of 48% and 84% compared to *4cl1*-*1* and *comt*-*4*, respectively. An additive effect was observed when an acid and an alkaline pretreatment was used (Additional file 3). With an acid pretreatment, an increase of 15% and 95% was observed in the double mutant compared to *4cl1*-*1* and *comt*-*4*, respectively. When an alkaline pretreatment was used, an increase of 9% and 42% was observed in the double mutant compared to *4cl1*-*1* and *comt*-*4*, respectively. If the glucose release is calculated per CWR, similar results were obtained (Additional files 4, 5, 6). Degradation of the primary inflorescence stem pieces into fibers was observed under acid and alkaline pretreatment for *4cl*-*1* and *4cl*-*1 comt*-*4* (Additional file 7).

Taken together, in almost all saccharification conditions (except *tra2 comt*-*1* without pretreatment), the double mutants had a significantly higher saccharification efficiency compared to the WT and the most efficient parental lines, which was even synergistic for *c4h3 comt*-*4* and *4cl1*-*1 comt*-*4* without pretreatment. Compared to the parental lines, the highest increase was observed for the *4cl1*-*1 comt*-*4* and *c4h*-*3 comt*-*4* double mutants when no pretreatment was used.

## Discussion

### Stacking different lignin traits does not always lead to a biomass yield penalty

In this study, we stacked reduced lignin content with G- and 5H-rich lignin by combining either *tra2*, *c4h*-*3,* or *4cl1*-*1* with a *comt* (*comt*-*1* or *comt*-*4*) mutation in Arabidopsis, to study the effect on saccharification efficiency. These mutants were specifically selected, because they do not display a growth reduction [41]. When the crosses of *c4h*-*3* and *4cl1*-*1* with *comt*-*4* were made, *comt*-*1* and *comt*-*4* were considered equivalent knockout mutants. However, later, experiments showed that the S/G ratio was more reduced in *comt*-*1* than in *comt*-*4* [8]. The cross between *tra2* and *comt*-*1* was added later to this study.

Stacking mutations in different lignin genes have already been attempted, but often resulted in plants that were dwarfed, even when the parental lines were not [53–57]. There are several factors that could lead to these growth defects, among others flux perturbations in the phenolic pathway. It has been shown that the general transcription regulator Mediator is involved in the response to disruptions in the phenylpropanoid pathway [60, 61]. Indeed, the *ref8*-*1* mutant (missense mutation in *p*-*coumaroylshikimate 3*-*hydroxylase* (*C3H*), Fig. 1) phenotype can be largely rescued by mutation of the genes encoding the Mediator complex subunits MED5a and MED5b. While growth, lignin level, and changes in gene expression in the phenylpropanoid pathway were largely restored, the synthesis of G and S units was not; the *med5a/5b ref8*-*1* triple mutant had an increased frequency of H units, just like the *ref8*-*1* mutant [60]. In addition, the Mediator plays a causative role in the reduced production of hydroxycinnamate esters and anthocyanins in *fah1* mutants [61]. These two studies support a model in which MED5a/5b is important for the homeostatic repression of phenylpropanoid metabolism [60, 61]. In addition, a strong reduction in the flux through the phenylpropanoid pathway and the consequent reduction in lignin deposition in the cell wall can result in a collapse of vessel cells, a phenotype called ‘irregular xylem’, resulting in a yield penalty [54, 62–65]. As a result of the collapsed vessels, plants cannot efficiently transport nutrients and water from the roots to the aerial parts of the plant [64]. Irregular xylem phenotypes have been observed in cellulose and hemicellulose mutants and in mutants with a drastic reduction in lignin content, while mutants with a milder reduction in lignin content generally do not show collapsed vessels [64, 66–70]. Perturbing two or more genes could also have more severe consequences on the phenotype than the individual mutated (or overexpressed) genes, because this could affect the flux through the phenolic pathway more dramatically.

Our study, however, demonstrates that it is possible to fine-tune the balance between lowering lignin content and altering its composition, resulting in healthy plants with an even higher saccharification efficiency than either of their parental lines. The resulting *tra2 comt*-*1*, *c4h*-*3 comt*-*4,* and *4cl1*-*1 comt*-*4* double mutants grew without measured growth defects. Importantly, the lignin mutants selected for this study do not block, but merely decrease the flux through certain steps in the lignin biosynthesis pathway. The T-DNA insertion lines *tra2* and *4cl1*-1 are knockout lines; however, because *TRA2* and *4CL1* are part of gene families, they most likely retain some functionality from the homologs *TRA1* and *4CL2*, *4CL3,* and/or *4CL4*, respectively [47]. Likewise, the line used to decrease the lignin amount by perturbing C4H, *c4h*-*3*, has a point mutation that likely retains partial C4H activity. Conversely, the *comt*-*1* and *comt*-*4* mutations almost completely block the flux to sinapyl alcohol, as judged from the low amount of S units in the lignin polymer. Taken together, in three independent stacking experiments, we have shown that the biomass yield can be maintained when reduced lignin levels are stacked with G and 5H rich lignin, and when the individual traits do not result in a biomass decrease.

### Stacking lignin traits increases the saccharification efficiency

The three double mutants studied (*tra2 comt*-*1*, *c4h*-*3 comt*-*4, and 4cl1*-*1 comt*-*4*) showed AcBr lignin amounts that were similar or even more reduced than those of the low-lignin parents *tra2*, *c4h*-*3,* and *4cl1*-*1* and an increased relative incorporation of 5H units, similar to the *comt* parental lines. However, the S/G ratio in *c4h*-*3 comt*-*4* and *4cl1*-*1 comt*-*4* was slightly higher compared to the parental line *comt*-*4* (but still strongly reduced compared to the WT, and *c4h*-*3* and *4cl1*-*1*, respectively). Most likely, this is due to the influence of the *c4h*-*3* and *4cl1*-*1* mutations, which cause a strong decrease in the lignin amount. Because F5H is the rate-limiting step, there might be a relative increase in S units when the flux through the phenylpropanoid pathway is reduced [71, 72]. Alternatively, the reduced flux through the phenylpropanoid pathway could cause feedback and feedforward mechanisms leading to an increase in S units [8, 41, 73].

Lignin amount and lignin composition are two factors that influence cell-wall recalcitrance towards saccharification [74]. The saccharification efficiency in the parental lines *tra2*, *c4h*-*3,* and *4cl1*-*1* is improved, most likely because of the lower lignin amount. In addition, the saccharification efficiency of *comt*-*1* and *comt*-*4* is improved. It has been proposed that the incorporation of 5H units in lignin reduces the cross-linking between lignin and polysaccharides in the cell wall. Quinone methides are produced as intermediates during the (cross-)coupling of the lignin monomers at their 8-positions, and intramolecular trapping of these quinone methides by the free 5-hydroxyls of the 5H lignin units would prevent covalent linkage of lignin with the polysaccharide hydroxyl groups [56, 74]. Reducing the interactions between hemicellulose and lignin loosens the matrix, which in turn improves the saccharification efficiency of the tissue. The increase in saccharification efficiency caused by G and 5H rich lignin is potentiated when combined with a low-lignin phenotype, as shown by the significant improvements displayed upon stacking of *tra2*, *c4h*-*3,* and *4cl1*-*1* with *comt*. Indeed, the saccharification efficiency increased upon acid and alkaline pretreatment in the *tra2 comt*-*1*, *c4h*-*3 comt*-*4,* and *4cl1*-*1 comt*-*4* double mutants compared to both their low-lignin parental lines and the *comt* parental line. The saccharification efficiency of the *c4h*-*3 comt*-*4* and *4cl1*-*1 comt*-*4* double mutants even increased synergistically when no pretreatment was used, roughly doubling the cellulose-to-glucose conversion as compared to WT.

Acid pretreatment makes the lignocellulosic biomass easier digestible mainly by dissolving the hemicellulose and partially pre-hydrolyzing the cellulose [75]. Alkaline pretreatment, on the other hand, loosens the lignin polymer [1, 76]. Although pretreatments improve the efficiency of the process, they are expensive and energy demanding, making them less attractive for the bio-refinery. Indeed, pretreatments can count up to 20% of the total capital investment needed [77]. Therefore, it is highly beneficial to produce easily degradable feedstock and decrease the requirement for pretreatments. The *c4h*-*3 comt*-*4* double mutant had an increase of 43% in saccharification efficiency compared to its most efficient parent *c4h*-*3* and in total 52% of all its cellulose was converted into glucose without pretreatment. The same trend was observed for the *4cl1*-*1 comt*-*4* double mutant, which displayed an increase of 47% in saccharification efficiency compared to its most efficient parent *4cl1*-*1*. This mutant line also had a 50% cellulose-to-glucose conversion with no pretreatment. Notably, both double mutants displayed saccharification efficiencies with no pretreatments comparable to those of their most efficient parents with acid pretreatment. Therefore, the stacking strategy has potential for the bio-refinery, because it permits the same cellulose-to-glucose conversion efficiencies to be reached without the need for costly pretreatments.

The ability to stack a low-lignin trait with a relative increase in G and 5H units (which individually do not negatively affect plant growth), without adversely affecting plant growth, and with a synergistic or additive effect on saccharification efficiency, is of interest for the bio-refinery. Further research will reveal whether the presented results can be translated to suitable biomass crops, such as sugarcane, maize, switchgrass, alfalfa, eucalyptus, or poplar. Although the effects seen upon lignin modification in Arabidopsis are often very similar to those in a crop, this is not always the case [36, 37, 48, 50, 78, 79]. Therefore, whether the obtained results can be translated to a bio-energy crop needs to be further investigated. Genome-editing techniques such as TALEN and CRISPR/Cas9 allow the creation of lignin double mutants in biomass crops. TALEN has already been successfully applied in sugarcane [37, 38] and maize [80]. CRISPR/Cas9 has already been successfully applied in maize [81], switchgrass [50, 82], and poplar [48, 83]. In poplar, mutations in the *4CL1* gene caused by CRISPR/Cas9 resulted in 25% less lignin, without a reported yield penalty [48]. These poplars can be used to validate the observations we made in Arabidopsis, e.g., by retransforming them with a gRNA targeting *COMT*.

## Conclusions

In this study, we stacked two different lignin traits (decreased lignin amount and lignin with an increased incorporation of G and 5H units) by crossing *tra2*, *c4h*-*3,* and *4cl1*-*1* with *comt*-*1* or *comt*-*4* in Arabidopsis. The three double mutants all had a reduced lignin amount, a lower S/G ratio, and an increased amount of 5H units, which means that the lignin traits of both parental lines were successfully combined in the double mutants. We found that the saccharification efficiency was improved in the double mutants, compared to the WT and the parental lines, without having a yield reduction of the primary inflorescence stem. The stacking of *c4h*-*3* and *4cl1*-*1* with *comt*-*4* led in both cases even to synergistic effects on the saccharification efficiency when the biomass was not pretreated. Our results show that combining two different lignin traits can improve saccharification efficiency without causing a biomass yield penalty.

## Methods

### Plant material, plant growth, and harvest

The *tra2*, *c4h*-*3*, *4cl1*-*1*, *comt*-*1,* and *comt*-*4* single mutants have been described before, all are in the Col-0 ecotype [8, 41, 42, 84]. The double mutants were obtained by crossing *tra2* with *comt*-*1*, *c4h*-*3* with *comt*-*4* and *4cl1*-*1* with *comt*-*4*. After self-pollination of the resulting heterozygous F1 offspring, homozygous plants were selected via PCR-based genotyping in the F2 generation. PCR conditions were as previously described [41]. Each homozygous double mutant was cultivated in soil with its corresponding control lines (*tra2 comt*-*1*: WT, *tra2* and *comt*-*1*; *c4h*-*3 comt*-*4*: WT, *c4h*-*3* and *comt*-*4*; and *4cl1*-*1 comt*-*4*: WT, *4cl1*-*1* and *comt*-*4*). To allow the development of a single tall inflorescence stem, plants were grown first for 6 weeks in short-day conditions (8-h light/16-h dark photoperiods, 21 °C, 55% humidity) after which they were transferred to long-day conditions (16-h light/8-h dark photoperiods, 21 °C, 55% humidity). When the plants were completely senesced and dry, the main stem was harvested just above the rosette. The siliques, axillary inflorescence, and rosette leaves were removed from the main stem and the height and mass of the stem were measured. The basal 30 cm (excluding the basal 1 cm) of the main stem were pooled two by two per genotype to obtain 10 biological replicates of each line. The stems were chopped in pieces of around 2 mm. These pooled samples were used for cell-wall analysis and saccharification assays.

### Lignin analysis

To obtain a purified CWR, the 10 pools of each line were subjected to a sequential extraction with water (98 °C), ethanol (76 °C), chloroform (59 °C), and acetone (54 °C) for 30 min each. The remaining CWR was dried under vacuum and the proportion of CWR per dry weight (% CWR) was calculated for each sample. The quantification of the lignin content was performed with an acetyl bromide method adapted for small sample sizes as previously described [85].

The composition of lignin was analyzed via thioacidolysis as previously described [86]. During thioacidolysis, monomers involved in β-*O*-4-ether bonds are released. Their trimethylsilyl ether derivates were analyzed through gas chromatography (GC) on a Hewlett-Packard HP6890 Series GC system (Agilent, Santa Clara, CA, USA) and detected with a coupled HP-5973 mass-selective detector. After peak integration, for quantification of the conventional lignin units H, G, and S, response factors as earlier reported were used [87]. For the 5H units, an average response factor was used [8].

### Cellulose quantification

Aliquots of 10 mg dry stem pieces were extracted to get the CWR, as described above. Cellulose content was estimated via the colorimetric Updegraff method as previously described [88]. The absorbance at 625 nm was measured at room temperature with a Spectramax 250 spectrophotometer (Sopachem, Brussels, Belgium).

### Saccharification assays

The saccharification assays were performed as previously described [8, 59]. Three aliquots of 10 mg dry 2-mm stem segments per pooled sample were used. An acid and alkaline pretreatment were performed on two of these aliquots (respectively, with 1 N HCl or 62.5-mM NaOH). The third aliquot was saccharified without any pretreatment. Glucose measurements were performed 3, 6, 24, 48, and 72, and if the plateau was not reached, 96 h after the saccharification enzymes were added. The enzyme activity added to each sample for *tra2 comt*-*1* was 0.023 FPU, for *c4h*-*3 comt*-*4* 0.019 FPU and for *4cl1*-*1 comt*-*4* 0.021 FPU. We defined the plateau as reached when the absolute increase in cellulose-to-glucose conversion was less than 2% (compared to the previous timepoint). Cellulose-to-glucose conversion was calculated based on the average released glucose/CWR and the average cellulose amount/CWR.

### Statistical analysis

All statistical analyses were performed with Rstudio 0.97.123 (Rstudio, 2009, Boston, Massachusetts). To test if the variables were normally distributed, the Shapiro–Wilks test was used. If the normality assumption was not fulfilled (*p* < 0.01), data were transformed. This was the case for the relative frequency of H, G, and S units and the S/G ratio in the experiment with *tra2 comt* double mutants. The applied transformations were as follows: HT = log (H); GT = 1/[log(G)]; ST = square root of (S); and S/G ratio T = square root of (S/G ratio). The homoscedasticity was assessed with the Levene’s test. Depending on these results, statistical analyses were performed with analysis-of-variance (ANOVA) using the lm function or with the Kruskal–Wallis test. Upon a significant ANOVA or Kruskal–Wallis test result (*p* < 0.05), the post hoc Holm method was performed (with pooled or non-pooled standard deviation, depending on the Levene’s test) to reveal which lines differed for the particular trait.

## Additional files

**Additional file 1.** Graphs of the cellulose-to-glucose conversions over time. Cellulose-to-glucose conversions of the *tra2 comt*-*1*, *c4h*-*3 comt*-*4,* and *4cl1*-*1 comt*-*4* double mutants, wild type, and the corresponding parental lines, without, with acid and with alkaline pretreatment at the different timepoints. **A**) Cellulose-to-glucose conversion for *tra2 comt*-*1* and its respective control lines. **B**) Cellulose-to-glucose conversion for *c4h*-*3 comt*-*4* and its respective control lines. **C**) Cellulose-to-glucose conversion for *4cl1*-*1 comt*-*4* and its respective control lines. The conversions were calculated based on the saccharification efficiency and cellulose content (both on CWR basis) and are expressed as % cellulose converted to glucose. Measurements were done 6, 24, 48, and 72 and eventually 96 h after the saccharification enzymes were added to the samples. The error bars represent standard deviations (*n* = 10). The exact values and significances are presented in Additional file 2. The relative increases in saccharification efficiency compared to wild type are presented in Additional file 3.

**Additional file 2.** Values and significances of the cellulose-to-glucose conversions over time. Cellulose-to-glucose conversions of the *tra2 comt*-*1*, *c4h*-*3 comt*-*4,* and *4cl1*-*1 comt*-*4* double mutants, wild type, and the corresponding parental lines (*n* = 10) (± SD), without, with acid, and with alkaline pretreatment at the different timepoints. Significance groups are indicated with the same letter in superscript and different letters represent significant differences at the 0.01 significance level (ANOVA, pairwise comparison with Holm correction).

**Additional file 3.** Relative increase in saccharification efficiency compared to wild type. The relative increase in saccharification efficiency of the parental lines and the double mutants compared to wild type was calculated based on the average of the cellulose-to-glucose conversion at the plateau. The variation was taken into account to determine whether there is an additive or synergistic effect.

**Additional file 4.** Graphs of the glucose release/CWR over time. Glucose release/CWR of the *tra2 comt*-*1*, *c4h*-*3 comt*-*4,* and *4cl1*-*1 comt*-*4* double mutants, wild type, and the corresponding parental lines, without, with acid and with alkaline pretreatment at the different timepoints. **A**) Glucose release/CWR for *tra2 comt*-*1* and its respective control lines. **B**) Glucose release/CWR for *c4h*-*3 comt*-*4* and its respective control lines. **C**) Glucose release/CWR for *4cl1*-*1 comt*-*4* and its respective control lines. Measurements were done 6, 24, 48, 72, and eventually 96 h after the saccharification enzymes were added to the samples. The error bars represent standard deviations (*n* = 10). The exact values and significances are presented in Additional file 5. The relative increases in saccharification efficiency compared to wild type are presented in Additional file 6.

**Additional file 5.** Values and significances of the released glucose/CWR over time. Released glucose/CWR of the *tra2 comt*-*1*, *c4h*-*3 comt*-*4* and *4cl1*-*1 comt*-*4* double mutants, wild type and the corresponding parental lines (*n* = 10) (± SD), without, with acid and with alkaline pretreatment at the different timepoints. Significance groups are indicated with the same letter in superscript and different letters represent significant differences at the 0.01 significance level (ANOVA, pairwise comparison with Holm correction).

**Additional file 6.** Relative increase in saccharification efficiency compared to wild type. The relative increase in saccharification efficiency of the parental lines and the double mutants compared to wild type was calculated based on the average of the released glucose/CWR at the plateau. The variation was taken into account to determine whether there is an additive or synergistic effect.

**Additional file 7.** Inflorescence stem pieces after saccharification. Inflorescence stem pieces of **A**) *tra2 comt*-*1*, **B**) *c4h*-*3 comt*-*4,* and **C**) *4cl1*-*1 comt*-*4* and their respective control lines after the plateau of saccharification was reached, without, with acid and with alkaline pretreatment. Bar 1 mm.
