# Supplementary figures and images for: Stacking of a low-lignin trait with an increased guaiacyl and 5-hydroxyguaiacyl unit trait leads to additive and synergistic effects on saccharification efficiency in Arabidopsis thaliana
Source: Biotechnol Biofuels. 2018 Sep 20;11:257. doi: 10.1186/s13068-018-1257-y (PMC6146604; doi:10.1186/s13068-018-1257-y)

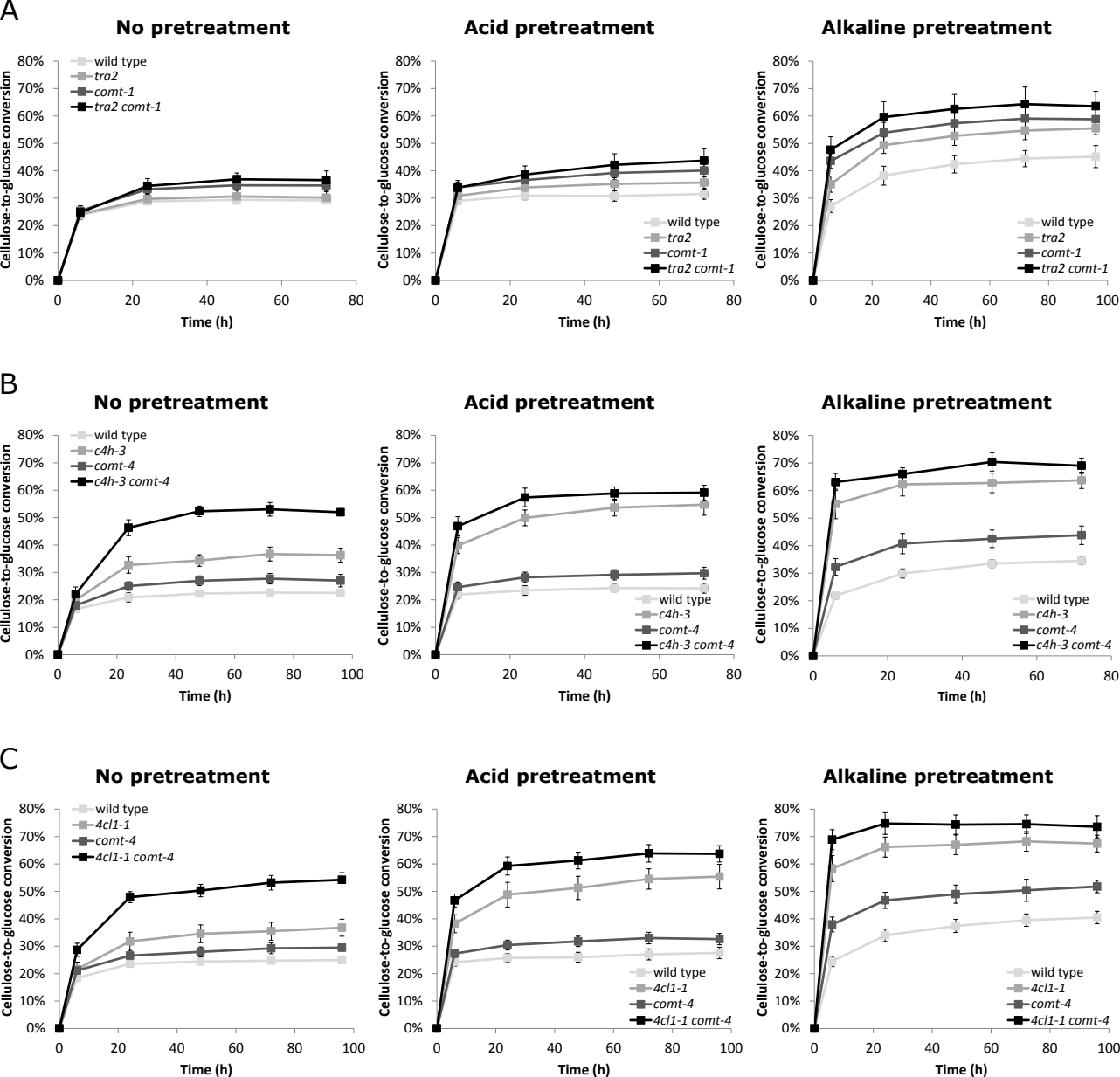

Supplement: Supplementary file 1 — Additional file 1. Graphs of the cellulose-to-glucose conversions over time. Cellulose-to-glucose conversions of the tra2 comt-1, c4h-3 comt-4, and 4cl1-1 comt-4 double mutants, wild type, and the corresponding parental lines, without, with acid and with alkaline pretreatment at the different timepoints. A) Cellulose-to-glucose conversion for tra2 comt-1 and its respective control lines. B) Cellulose-to-glucose conversion for c4h-3 comt-4 and its respective control lines. C) Cellulose-to-glucose conversion for 4cl1-1 comt-4 and its respective control lines. The conversions were calculated based on the saccharification efficiency and cellulose content (both on CWR basis) and are expressed as % cellulose converted to glucose. Measurements were done 6, 24, 48, and 72 and eventually 96 h after the saccharification enzymes were added to the samples. The error bars represent standard deviations (n = 10). The exact values and significances are presented in Additional file 2. The relative increases in saccharification efficiency compared to wild type are presented in Additional file 3. [file 13068_2018_1257_MOESM1_ESM.pdf]

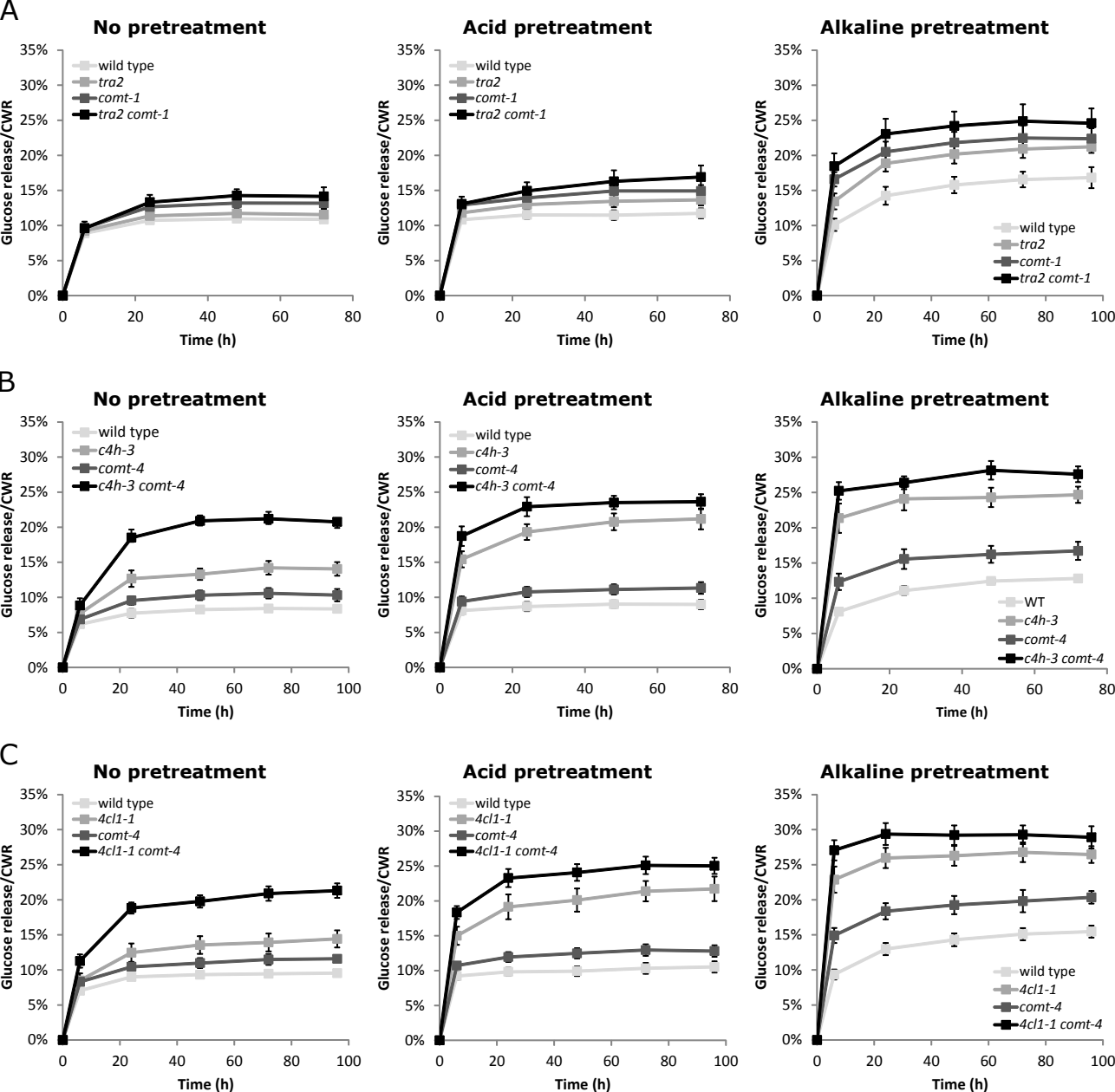

Supplement: Supplementary file 4 — Additional file 4. Graphs of the glucose release/CWR over time. Glucose release/CWR of the tra2 comt-1, c4h-3 comt-4, and 4cl1-1 comt-4 double mutants, wild type, and the corresponding parental lines, without, with acid and with alkaline pretreatment at the different timepoints. A) Glucose release/CWR for tra2 comt-1 and its respective control lines. B) Glucose release/CWR for c4h-3 comt-4 and its respective control lines. C) Glucose release/CWR for 4cl1-1 comt-4 and its respective control lines. Measurements were done 6, 24, 48, 72, and eventually 96 h after the saccharification enzymes were added to the samples. The error bars represent standard deviations (n = 10). The exact values and significances are presented in Additional file 5. The relative increases in saccharification efficiency compared to wild type are presented in Additional file 6. [file 13068_2018_1257_MOESM4_ESM.pdf]
