# Supplementary material for: Stacking of a low-lignin trait with an increased guaiacyl and 5-hydroxyguaiacyl unit trait leads to additive and synergistic effects on saccharification efficiency in Arabidopsis thaliana
Source: Biotechnol Biofuels. 2018 Sep 20;11:257. doi: 10.1186/s13068-018-1257-y (PMC6146604; doi:10.1186/s13068-018-1257-y)
Supplement: Supplementary file 7 — Additional file 7. Inflorescence stem pieces after saccharification. Inflorescence stem pieces of A) tra2 comt-1, B) c4h-3 comt-4, and C) 4cl1-1 comt-4 and their respective control lines after the plateau of saccharification was reached, without, with acid and with alkaline pretreatment. Bar 1 mm. [file 13068_2018_1257_MOESM7_ESM.pdf]

A

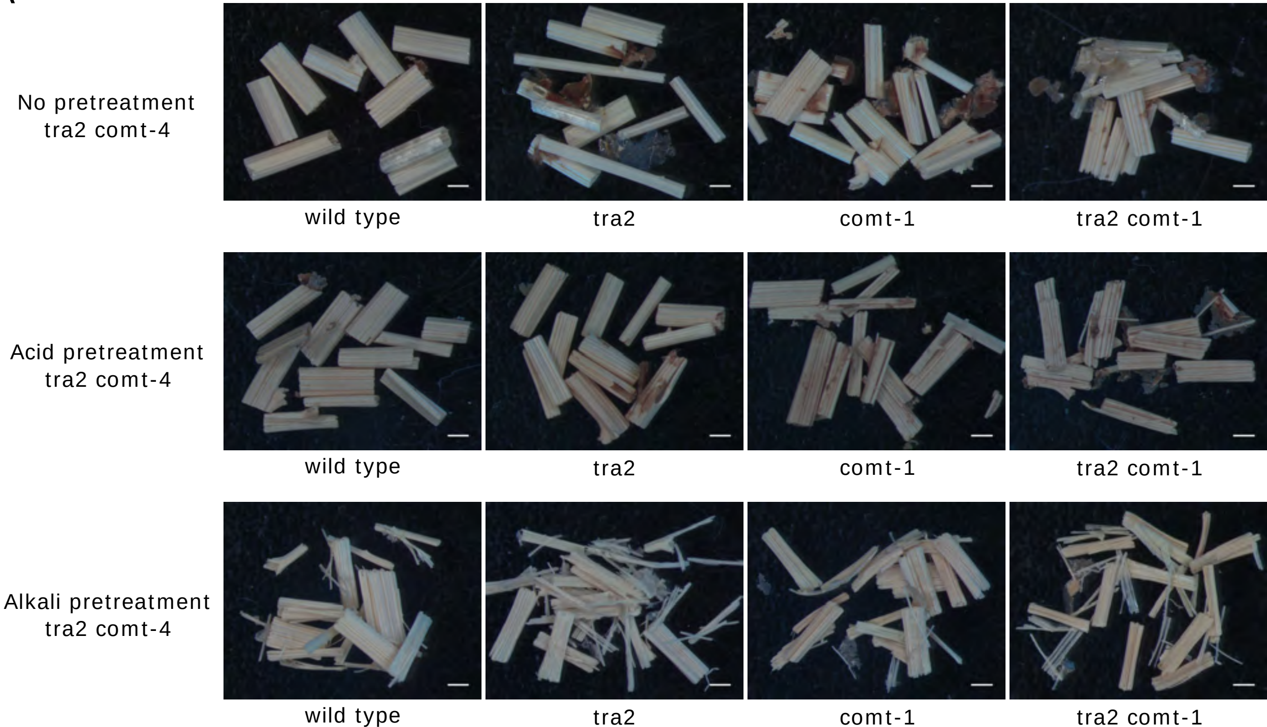
No pretreatment
tra2 comt-4
Acid pretreatment
tra2 comt-4
Alkali pretreatment
tra2 comt-4
wild type
tra2
comt-1
tra2 comt-1
wild type
tra2
comt-1
tra2 comt-1
wild type
tra2
comt-1
tra2 comt-1

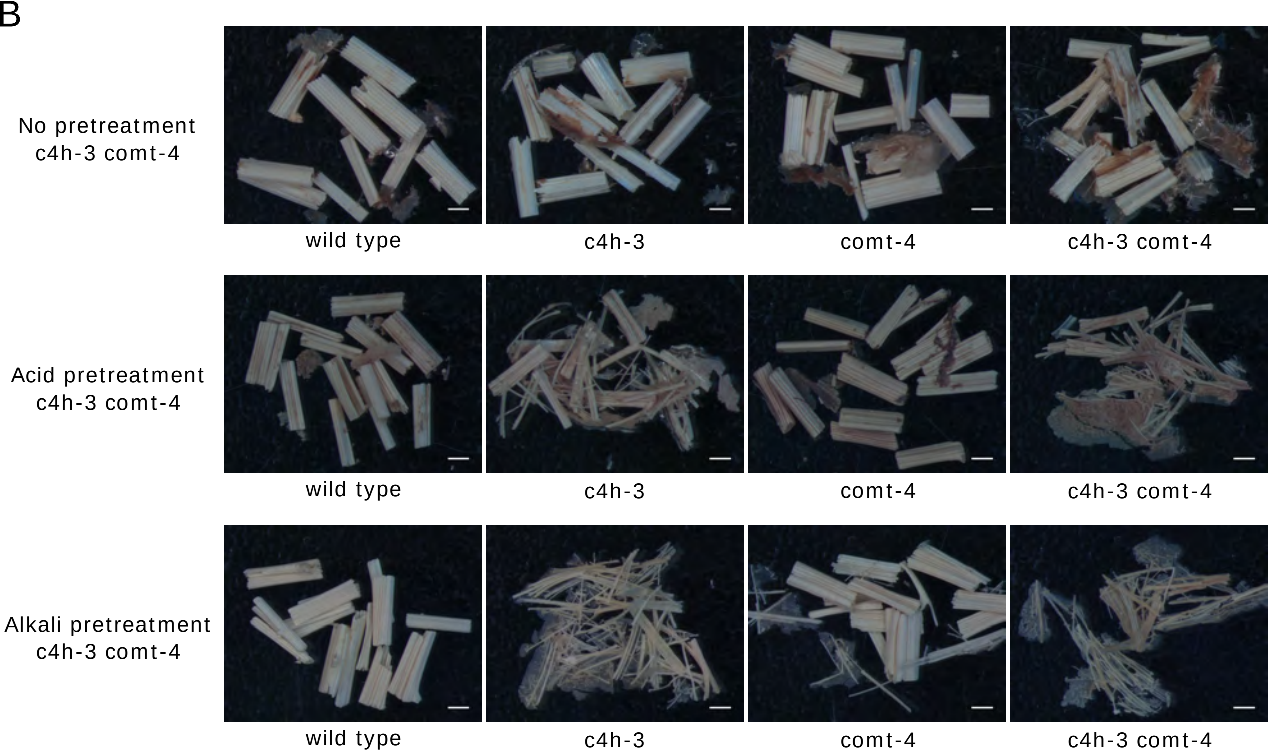
B
No pretreatment c4h-3 comt-4
Acid pretreatment c4h-3 comt-4
Alkali pretreatment c4h-3 comt-4
wild type
c4h-3
comt-4
c4h-3 comt-4

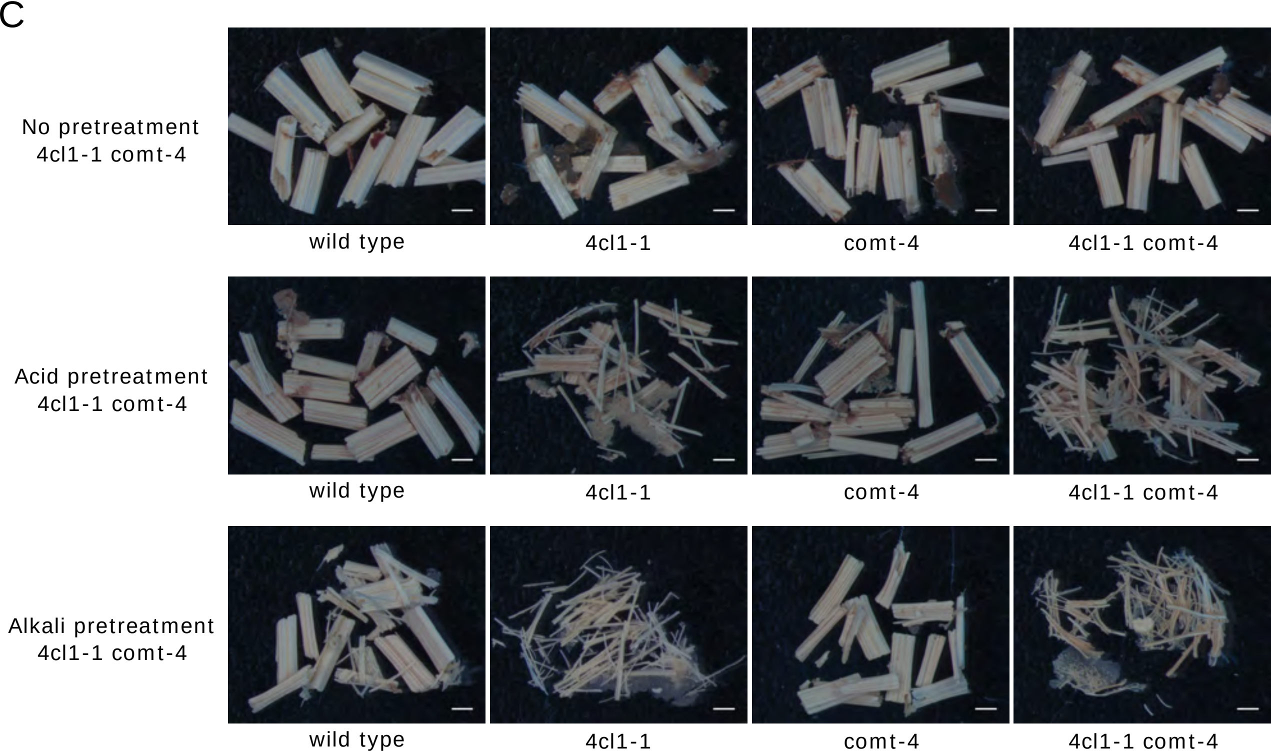
C
No pretreatment
4cl1-1 comt-4
wild type
4cl1-1
comt-4
4cl1-1 comt-4
Acid pretreatment
4cl1-1 comt-4
wild type
4cl1-1
comt-4
4cl1-1 comt-4
Alkali pretreatment
4cl1-1 comt-4
wild type
4cl1-1
comt-4
4cl1-1 comt-4
